# Characteristic analysis of TCR β-chain CDR3 repertoire for pre- and post-liver transplantation

**DOI:** 10.18632/oncotarget.26138

**Published:** 2018-10-02

**Authors:** Guiqi Yang, Minglin Ou, Huaizhou Chen, Changchun Guo, Jiejing Chen, Hua Lin, Donge Tang, Wen Xue, Wenlong Li, Weiguo Sui, Yong Dai

**Affiliations:** ^1^ Guangxi Key Laboratory of Metabolic Diseases Research, Guilin 541002, P.R. China; ^2^ Clinical Medical Research Center, The Second Clinical Medical College of Jinan University, Shenzhen, Guangdong 518020, P.R. China; ^3^ The Pingshan People’s Hospital of Shenzhen, Shenzhen, Guangdong 518118, P.R. China; ^4^ The Technology Company of iCarbonX, Shenzhen, Guangdong 518000, P.R. China

**Keywords:** T cell receptor, immune repertoire, next-generation sequencing, liver transplantation

## Abstract

Liver cirrhosis of hepatitis B is an immune-related disease in which liver cells die during the body’s immune system activation to clear the virus, and the progress is closely related to T lymphocytes. T lymphocyte cells recognise antigens, specifically by major histocompatibility complex (MHC), through a membrane protein T cell receptor (TCR). Here, we used high throughput immune repertoire sequencing technique to study the characteristics and diversity of the TCR repertoire between patients who underwent liver transplantation and healthy controls (NC). We sequenced the TCR β-chain complementary-determining region 3 (CDR3) repertoire in peripheral blood mononuclear cells (PBMCs) from 6 liver transplantation patients before transplantation (Pre) and on the first (Post1) and seventh days (Post7) after transplantation along with 6 NC. We observed that the distributions of CDR3, VD indel, and DJ indel lengths were similar among the Pre, Post1, Post7 and NC groups. We found that the TCR repertoire diversity of transplantation groups was relatively lower compared to NC group. The Pre-group had more highly expanded T cell clones compared to Post1, Post7 and NC groups, and the diversity of the T cell repertoire of the Post7 group was significantly decreased compared to the Pre, Post1 and NC groups. In addition, we found our results also show that various TRBV expression increased and some public sequences at different time points after liver transplantation, and the expression levels of 3 TRBV segments and 2 TRBJ segments were also significantly different in Pre, Post1, Post7 and NC groups. Moreover, 1 aa sequence shared by all liver transplantation patients and 2 aa sequences shared by at least two groups, which may serve as biomarkers to monitor the immune status of liver transplant patients.

## INTRODUCTION

Chronic hepatitis B virus (HBV) infection leads to a range of liver diseases with a series of symptoms. From chronic hepatitis, complications of liver cirrhosis to hepatocellular carcinoma (HCC) are observed, which it is a major global public health problem [1]. HBV infection causes 500,000 to 1.2 million deaths every year, 320,000 of which are due to HCC [2]. The prognosis for patients with decompensated HBV cirrhosis is poor, with a 5-year survival of only 14% compared with 84% in patients with compensated HBV cirrhosis [3].

The ultimate cure for end-stage liver disease is liver transplantation, but many patients with advanced hepatitis B worldwide do not have access to or are not eligible for this treatment modality [4–5]. Liver function disorder of decompensated hepatitis B cirrhosis results in a series of body disorders, in turn affecting the body’s immune system and breaking up the diversity of the immune cells, eventually leading to the imbalance of the immune system. T lymphocytes are the key mediators of adaptive immunity, the invasion of pathogens, rejection and immune tolerance after organ transplantation. There are no accurate indicators of immune function after liver transplantation. Therefore, establishing the immune repertoire of decompensated hepatitis B cirrhosis patients during the perioperative period in liver transplantation is very urgent and important, as it may guide the rational application of individualised immunosuppressive drugs. This will further elucidate the aetiology and pathogenesis of decompensated hepatitis B cirrhosis, deepen the understanding of the disease aetiology, and provide a new opportunity for disease prevention, diagnosis and treatment. The immune repertoire (IR) refers to all functional B cells and T cells in the circulatory system at any time point [6]. T lymphocyte cells recognise antigens specifically by MHC through a membrane protein T cell receptor (TCR), which plays an important role in protection against graft-versus-leukaemia, opportunistic infections and other clinical challenges during early organ transplantation [7–8]. In general, T cells develop in the thymus from progenitors originating from haematopoietic stem cells in the bone marrow, and every T cell encodes a unique TCR. TCRs are heterodimeric, and approximately 90%–95% consist of an α chain and β chain, and approximately 5%–10% consist of a γ chain and δ chain. Each chain can be divided into variable regions (V region), cytoplasmic domains, consistent regions (C region) and transmembrane domains. The V region of the α chain and β chain has three hypervariable regions called complimentary- determining regions (CDRs): CDR1, CDR2 and CDR3, among which CDR3 is the most variable and directly determines the antigen-binding specificity of TCRs. In the maturity process of lymphocytes, CDR3 undergoes genomic rearrangement among the Variable (V), Diversity (D) and Joining (J) gene segments, plus the addition/subtraction of non-templated bases at recombinant junctions. These events finally contribute to the diversity of T cells, and different T cell clones have different CDR3 sequences [9–10]. A study predicted that the number of diverse TCR αβ pairs has been found up to 2.5 × 10^17^, which makes the T cell repertoire difficult to analyze [11]. The traditional techniques (such as flow cytometry and immune scanning spectrum analysis technique) cannot analyze the overall repertoire [12]. The development of new generation sequencing technology, including next-generation sequencing (NGS) technologies, in which sequences are decoded on arrays and millions of sequences can be read simultaneously [13], has been transformative for immune repertoire analysis.

Here, we evaluated the diversity of the immune system per patient with hepatitis B in decompensated liver cirrhosis before transplantation (Pre) and at two time points after liver transplantation (on the first (Post1) and seventh days (Post7) after transplantation) in a cohort of 6 patients compared to healthy controls (NC, *n =* 6) by amplifying the CDRs of TCRs using multiplex-PCR, then exploring the association between the immune repertoire and disease by high-throughput sequencing. The large-scale sequencing of the immune receptor repertoire offers a distinct and highly detailed molecular characterisation that may reform our perception of the immune system. Moreover, this method allows for the identification of the receptors expressed by expanded clonal T cell populations, which may be associated with the immune pathogenesis of donor antigens, and provides the basis for further investigation into the mechanisms of rejection during the perioperative period in liver transplantation. Based on these studies, we can delineate the immune repertoire of liver transplant patients, which might help in the evaluation of the preoperative and postoperative immune system and help to induce tolerance selectively against transplantation antigens. Furthermore, this study can offer important new information for the classification and monitoring of liver transplant recipients.

## RESULTS

We sequenced TCR repertoires collected from the peripheral blood of six healthy individuals (NC) and six patients of hepatitis B with decompensated liver cirrhosis (including Pre (*n =* 6), Post1 (*n =* 6), Post7 (*n =* 6) groups) by high-throughput sequencing (Illumina Genome Analyzer), obtaining an average of 18059673.79 total raw reads in each sample. Then, the raw reads were filtered, including adapter contamination, removal of low-quality reads and bases with low quality (lower than 10), and used miTCR, which was developed by MiLaboratory [14], to map the reads to a database. The TCRβ CDR3 sequences were identified. We collected an average of 15844686.04 reads per sample, which met the quality requirements after alignment. The total sequences, total input sequences, and unique CDR3 amino acid total clonotypes of each sample are shown in Table 1.

**Table 1:** TCRβ sequence statistics of all samples

sample	Total reads (pair)	Filter rate (%)	All reads number	Total input sequences	Total good sequences	Clones	Out of frame clones (%)
NC-1	14531938	7.58	13429915	12741793	6974774	65236	28.95
NC-2	15120523	5.60	14279961	13686795	9294862	76888	32.86
NC-3	11705732	2.27	11439695	10962971	9413701	89202	39.45
NC-4	20742618	3.41	20035882	19624585	16349931	75953	42.97
NC-5	21233023	4.22	20336693	19905248	12191501	93477	37.25
NC-6	23280243	3.11	22556798	2726023	2452132	75408	21.62
pre-1	14123714	16.67	11769844	11561054	9234279	55901	25.8
pre-2	14449375	4.58	13786945	13558505	10477547	96471	31.58
pre-3	17335420	16.57	14463087	14160116	11007669	36780	29.87
pre-4	14342880	9.38	12998104	12779055	2631309	9247	29.45
pre-5	16429473	14.52	14043757	13789324	3869428	8294	27.65
pre-6	22316233	6.00	20976527	20190190	17321956	70954	41.26
Post1-1	17567602	24.89	13194638	12819664	6888316	16438	27.0
Post1-2	14191447	14.87	12081718	11868324	5623017	18603	27.68
Post1-3	16328862	10.10	14679046	14489682	7864389	45344	24.72
Post1-4	14312776	15.10	12151499	11891015	9959463	47491	33.18
Post1-5	17246806	38.69	10574720	10094499	8585701	17373	21.63
Post1-6	23301649	8.53	21314090	20534392	11390657	27946	36.97
Post7-1	14771074	4.99	14034535	13885138	12628614	186033	31.48
Post7-2	14209864	5.34	13450561	13307342	11461755	62653	30.33
Post7-3	36614213	31.98	24906707	24331925	14800101	44186	25.21
Post7-4	15083842	6.45	14111126	13898803	11983390	120267	36.56
Post7-5	18531630	16.27	15517186	15254872	10407446	20085	36.21
Post7-6	25658834	5.92	24139431	23083948	15498797	46833	40.31

### Shared TCR clones among subjects

We assessed how commonly TCR sequences were shared among the different subjects of the same groups. We used high-throughput sequencing to characterise the corresponding portions of the respective TCR repertoires from the Pre-group, Post1 group, Post7 group and NC group. We further assessed how commonly TCR sequences were shared between different individuals of the same group. Approximately 4.68 × 10^5^, 3.12 × 10^5^, 5.45 × 10^5^ and 9.14 × 10^5^ unique CDR3 nucleotide sequences were obtained for the TCR repertoires of the pre-group, post1 group, post7 group and NC group, respectively, which corresponded to approximately 4.83 × 10^5^, 2.82 × 10^5^, 4.21 × 10^5^ and 8.02 × 10^5^ unique TCR amino acid(aa) sequences, respectively. We combined all the data for each group for further analysis and then binned unique CDR3 DNA sequences and aa sequences based on the number of subjects in which they occurred. We found almost all the CDR3 DNA sequences and aa sequences were found in only one subject. A small number of sequences were shared among the individuals.

Another interesting finding in our study was that 30 amino acid sequences and 15 CDR3 DNA sequences were observed in one healthy individual and also observed in other healthy individuals, and 1 amino acid sequence was observed in all the patients (Pre-group, Post1 and Post7 group). For example, the amino acid sequence CASSLGGSYEQYF was presented in all patients (Pre-group, Post1 and Post7 group), but not in control samples. Furthermore, we also found that 1 amino acid sequence was shared by the Post1 and Post7 groups and control samples. However, the amino acid sequence CASSLGETQYF was observed in Post1 and Post7 groups and all control samples. We next inquired into the possible public TCRs, which were shared in liver cirrhosis of hepatitis B pathogenesis. We analysed the CDR3 DNA and amino acid sequences of all the public in all the subjects, and a summary is shown in Supplementary Table 2. The function of the highly expanded clones (HECs) and amino acid sequences have not been verified. Identification of the liver cirrhosis-specific public TCR CDR3 DNA and amino acid sequences or the HECs of the control and diseases groups (Pre-group, Post1 and Post7group) may help us in understanding antigen selection and may also be critical for us to understand the immunoregulation of T cells for liver cirrhosis pathogenesis.

### Distribution characteristics of CDR3 length, VD indel length and DJ indel length references

**Figure 1:**
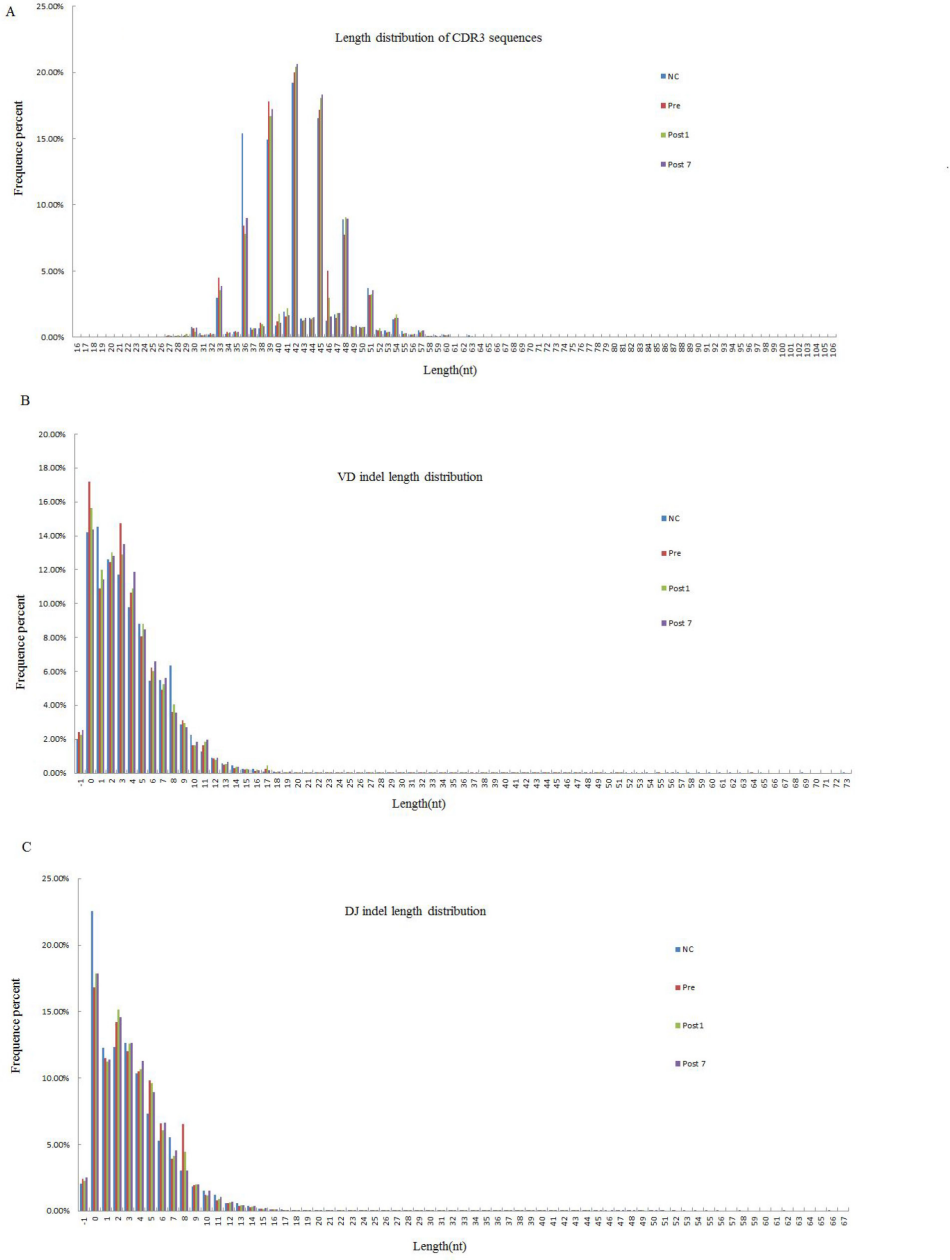
The Length distribution of CDR3, VD indel, and DJ indel in the NC group (*n* = 6), Pre group (*n* = 6), Post1 group (*n* = 6) and Post7 group (*n* = 6) (average of six individuals) The length distributions of CDR3 appear to be consistent in Pre、Post1、Post7 and NC group. (**A**) Of the 238,310,735 total good sequences for among the 4 groups, and the 5 most frequently observed CDR3 lengths were were 42, 45, 36, 39 and 48 nt. (**B**) The Total number of (inserted ± deleted) nucleotides at Vβ-Dβjunctions. The 5 most frequently observed VD indel lengths were 0, 2, 3, 1 and 4 nt. (**C**) Total number of (inserted ± deleted) nucleotides at Dβ-Jβ junctions. The 5 most common observed DJ indel lengths were the same as VD indel lengths, which also were 0, 2, 3, 1 and 4 nt.

We can obtain the information about the last position of the V gene on the sequence, the start position of the D gene, the last site of the D gene and the start site of the J gene after alignment and structural analysis. According to the loci information, to find the bases of indel V-D-J recombination, statistical analysis and plotting its length distribution is necessary. Moreover, the TCR CDR3 region of sequences was determined, and the CDR3β length distribution, VD indel length and DJ indel length are shown in Figure 1. As observed in Figure 1, CDR3 varied from 16 to 106 nt, with a peak at 42 in each group, and the 5 most frequently observed CDR3 lengths were 42, 45, 36, 39 and 48 nt. The VD indel length ranged from −1 to 73 nt, and the DJ indel length ranged from −1 to 68 nt. We found that the CDR3 length distribution, VD indel length and DJ indel length were similar in controls and patients with hepatitis B in decompensated liver cirrhosis (including Pre, Post1 and Post7 groups).

### Degree of expansion and impact of T cell clones

**Figure 2:**
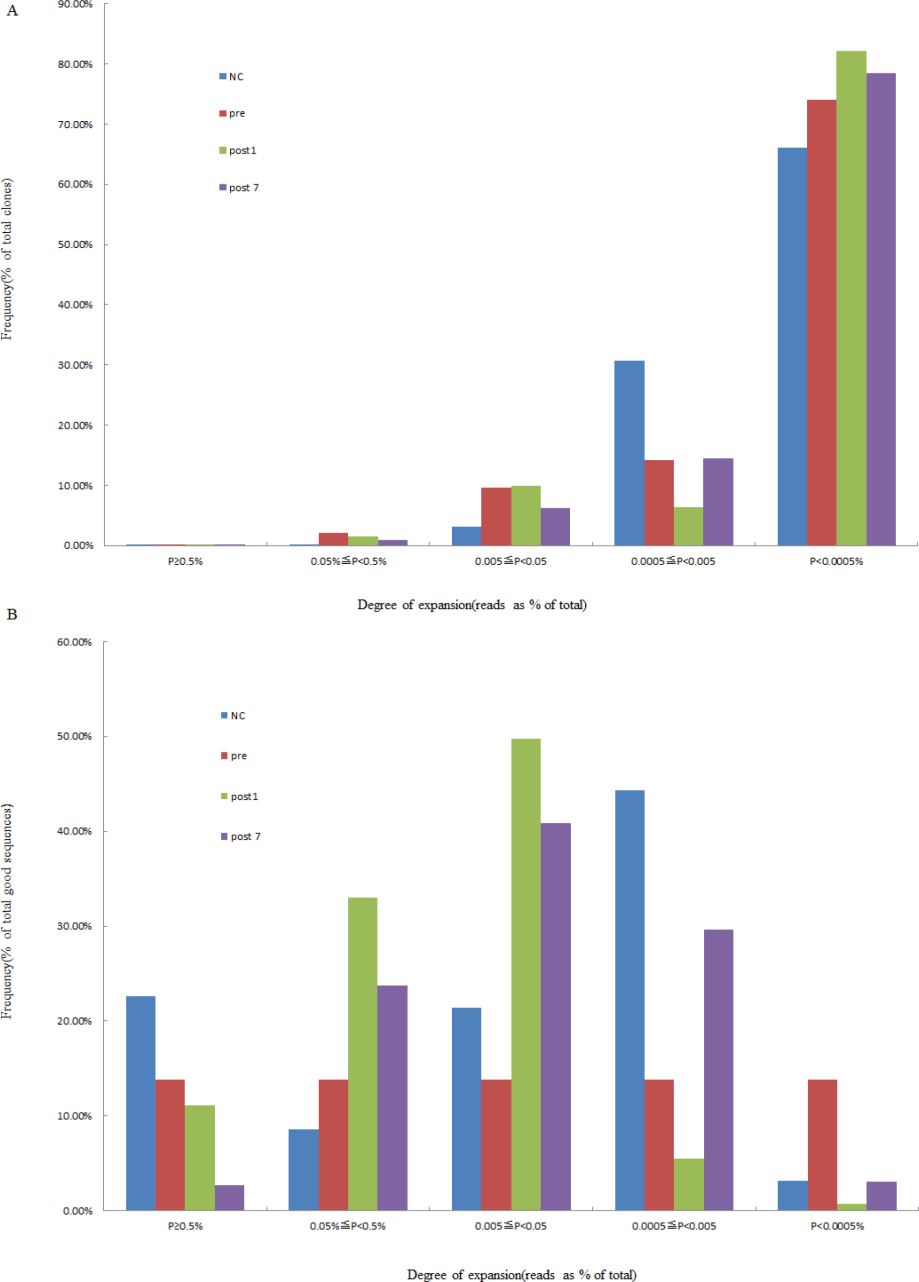
Degree of expansion and frequency distribution of T cell clones (**A**) The frequency distribution showed that the majority of the clones were low frequency. (**B**) The percentage of frequency distribution showed that the majority of the repertoire was comprised of a small number of HECs. The x-axis depicts the degree of expansion; the y-axis depicts the percentage of each clone frequency in total clones (or total good sequence).

According to the identity of each sequence after alignment, we calculated the frequency of expression of each clone. Then, the degree of expansion of each individual clone was estimated, which was based on the unique CDR3 sequence frequency within a sample and divided into 5 groups (≥0.5%, 0.05∼0.5%, 0.005∼0.05%, 0.0005∼0.005%, and <0.0005%, respectively). We defined that TCR clones with a frequency more than 0.5% of total reads in a sample were HECs. After statistical analyses, we found that only 1.11 × 10^−2^ (range, 4.48 × 10^−3^ to 2.30 × 10^−2^) of the clones were expanded beyond this threshold in the healthy control group. This corresponded to a median of 8.3 clones (range 4 to 15), in absolute numbers. In the Pre-group, there were 7.2 HECs (range, 1 to 16), with a proportion of 4.99 × 10^–2^ (range 2.72×10^−3^ to 1.73 × 10^−1^) in total clones. We also observed that 3.35×10^–2^ (range, 4.41 × 10^−3^ to 7.95 × 10^−2^) and 3.86 × 10^−3^ (range 1.08 × 10^−3^ to 7.98 × 10^−3^) of the clones were HECs in the Post1 and Post7 groups, and with absolute numbers, a median of 6.8 clones (median, 2 to 13) and 2.3 clones (median, 1 to 5) in the Post1 and Post7 group (Table 2 and Figure 2). Comparison of the degree of expansion of the most expanded clones in each group revealed that the clones in the Post7 groups were significantly more than in other groups (*P* = 0.046). We also found that a comparison of the degree of expansion of the most expanded the total good sequences in the Post7 group were significantly more expanded than in the NC group (*P* = 0.026). Afterwards, we determined the contribution of HECs to the whole T cell TCR repertoire and found that most of the repertoires consisted of a small number of HECs. For the Pre-group, there were 43 HECs (4.99 × 10^−2^% of all the unique CDR3 sequences) that accounted for 13.79% of the T cell sequences (total good reads); however, 74.01% of the clones were very low-frequency clones (<0.0005% of the total TCR sequences analysed), which accounted for only 1.42% of the T cell sequences that were present (total good reads).

**Table 2:** Comparison of the clonotype abundance distribution in each group among unique TCRβ nucleotide clonotypes and across the total TCRβ nucleotide repertoires

Degree of expansion	Clones (%)			Total good sequences		
	Pre group	NC group	*P*	NC group	Pre group	*P*
≥0.5%	4.99 × 10^−2^ ± 7.03 × 10^−2^	1.11 × 10^−2^ ± 6.68 × 10^−3^	0.251	22.59 ± 14.72	13.79 ± 10.41	0.293
0.05%≦-<0.5%	2.16 ± 3.292 × 10^−2^	7.55 × 10^−2^ ± 3.2	0.180	8.59 ± 2.60	23.79 ± 28.04	0.238
0.005≦-<0.05	9.58 ± 3.81	3.20 ± 2.50	0.002	21.41 ± 17.03	43.03 ± 22.21	0.132
0.0005≦-<0.005	14.21 ± 8.66	30.65 ± 9.86	0.007	44.29. ± 10.86	17.97 ± 15.96	0.014
<0.0005%	74.01 ± 4.65	66.07 ± 8.11	0.066	3.11 ± 1.65	1.42 ± 1.31	0.098

Degree of expansion	Clones (%)			Total good sequences		
	Pre group	post1 group	*P*	Pre group	post1 group	*P*
≥0.5%	4.99 × 10^−2^ ± 7.03 × 10^−2^	3.35 × 10^−2^ ± 3.04 × 10^−2^	0.626	13.79 ± 10.41	11.09 ± 11.65	0.541
0.05%≦-<0.5%	2.16 ± 3.29	1.57 ± 1.33	0.676	23.79 ± 28.04	32.97 ± 29.17	0.365
0.005≦-<0.05	9.58 ± 3.81	9.85 ± 4.44	0.911	43.03 ± 22.21	49.75 ± 27.91	0.360
0.0005≦–-<0.005	14.21 ± 8.66	6.36 ± 7.48	0.215	17.97 ± 15.96	5.45 ± 8.42	0.210
<0.0005%	74.01 ± 4.65	82.19 ± 9.33	0.104	1.42 ± 1.31	7.45 × 10^−1^ ± 3.95 × 10^−1^	0.318

Degree of expansion	Clones (%)			Total good sequences		
	Pre group	post7 group	*P*	Pre group	post7 group	*P*
≥0.5%	4.99 × 10^−2^ ± 7.03 × 10^−2^	3.86 × 10^−3^ ± 2.5 × 10^−3^	0.172	13.79 ± 10.41	2.67 ± 1.80	0.049
0.05%≦-<0.5%	2.16 ± 3.29	8.85 × 10−1 ± 1.36	0.290	23.79 ± 28.04	23.78 ± 28.25	0.999
0.005≦-<0.05	9.58 ± 3.81	6.27 ± 5.17	0.345	43.03 ± 22.21	40.87 ± 26.02	0.879
0.0005≦-<0.005	14.21 ± 8.66	14.43 ± 12.10	0.967	17.97 ± 15.96	29.61 ± 31.69	0.438
<0.0005%	74.01 ± 4.65	78.41 ± 9.98	0.358	1.42 ± 1.31	3.05 ± 3.74	0.309

Degree of expansion	Clones (%)			Total good sequences		
	post1 group	post7 group	*P*	post1 group	post7	*P*
≥0.5%	3.35 × 10^−2^ ± 3.04 × 10^−2^	3.86 × 10^−3^ ± 2.5 × 10^−3^	0.046	11.09 ± 11.65	2.67 ± 1.80	0.148
0.05%≦-<0.5%	1.57 ± 1.33	8.85 × 10−1 ± 1.36	0.330	32.97 ± 29.17	23.78 ± 28.25	0.535
0.005≦-<0.05	9.85 ± 4.44	6.27 ± 5.17	0.217	49.75 ± 27.91	40.87 ± 26.02	0.520
0.0005≦-<0.005	6.36 ± 7.48	14.43 ± 12.10	0.093	5.45 ± 8.42	29.61 ± 31.69	0.094
<0.0005%	82.19 ± 9.33	78.41 ± 9.98	0.309	7.45 × 10^−1^ ± 3.95 × 10^−1^	3.05 ± 3.74	0.186

**Figure 3:**
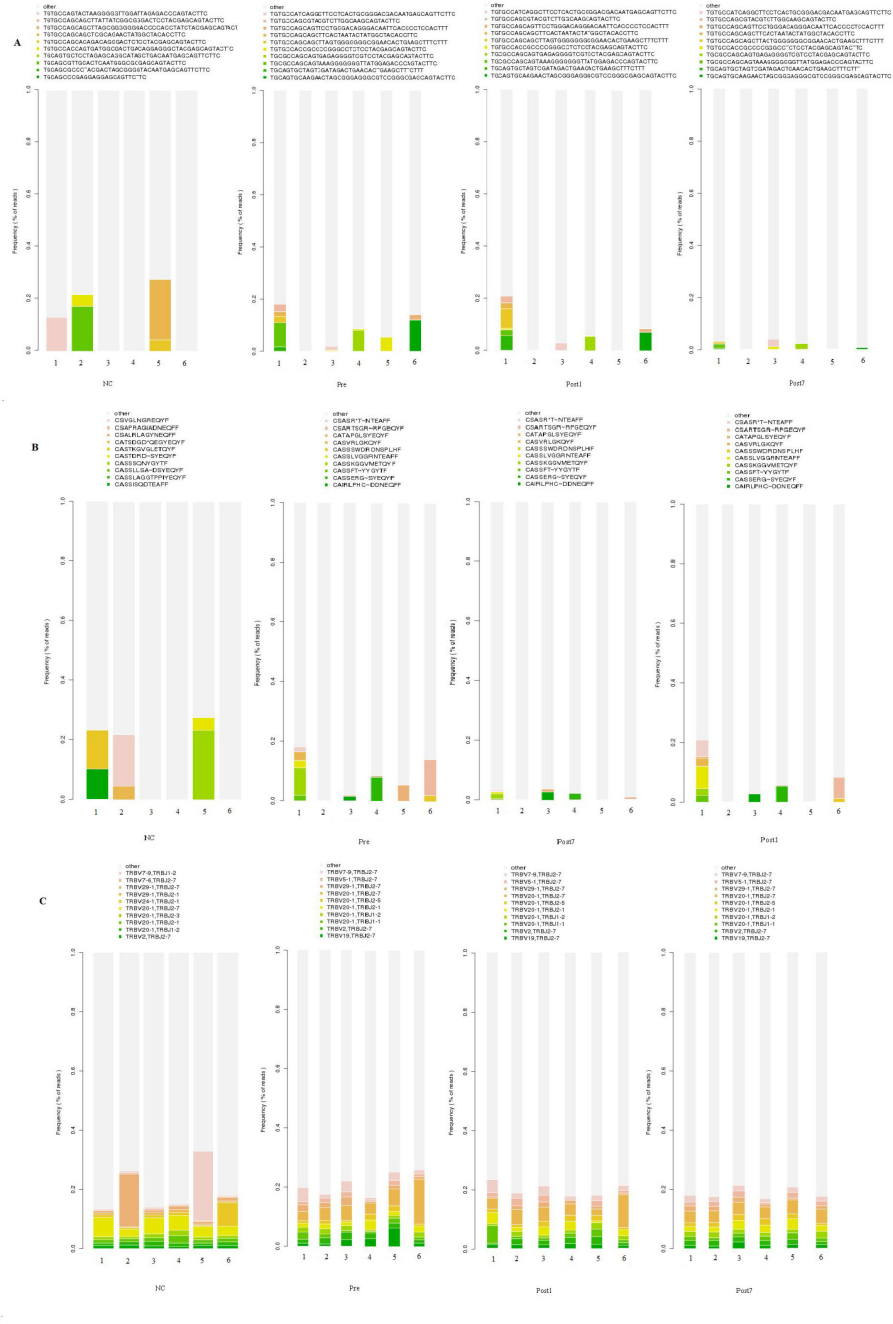
Quantification of T cell repertoire across the Pre、Post1、Post7 group and NC group The ten most common unique TCR βCDR3 sequences (**A**), CDR3 amino acids (**B**), and V-J combinations (**C**) in the different groups (6 individuals in each group) are indicated in colors, and the remaining clonotypes are grouped together in gray.

As described above, there were 50 HECs (1.11 × 10^−2^ ± 6.68 × 10^−3^% of the total clones) in the NC group, which was more than that of the Pre-group (43 HECs, accounted for 4.99 × 10^−2^ ± 7.03 × 10^−2^% of the total clones), in the Post1 group (41 HECs, accounted for 3.35 × 10^−2^ ± 3.04 × 10^−2^% of the total clones) and in the Post7 group (14 HECs, 3.86 × 10^−3^ ± 2.5 × 10^−3^% of the total clones). The number of HEC sequences in the Post7 group was the least among the groups. We have summarised these HECs in Supplementary Table 1. Then, we investigated whether the HEC sequences overlapped between the different groups and found that there were no HECs shared among the groups. The 10 most expanded clones were plotted to compare the differences in DNA sequences, amino acid levels, and V-J combinations in each group (Figure 3). From Figure 3, the findings did not provide strong evidence of similarities of HECs among the different subjects at the DNA sequence and amino acid levels. However, the ten most common V–J combinations were observed in all subjects (including normal individuals and the liver transplant recipients with hepatitis B cirrhosis of the liver decompensation); only the frequency distribution was inconsistent.

**Figure 4:**
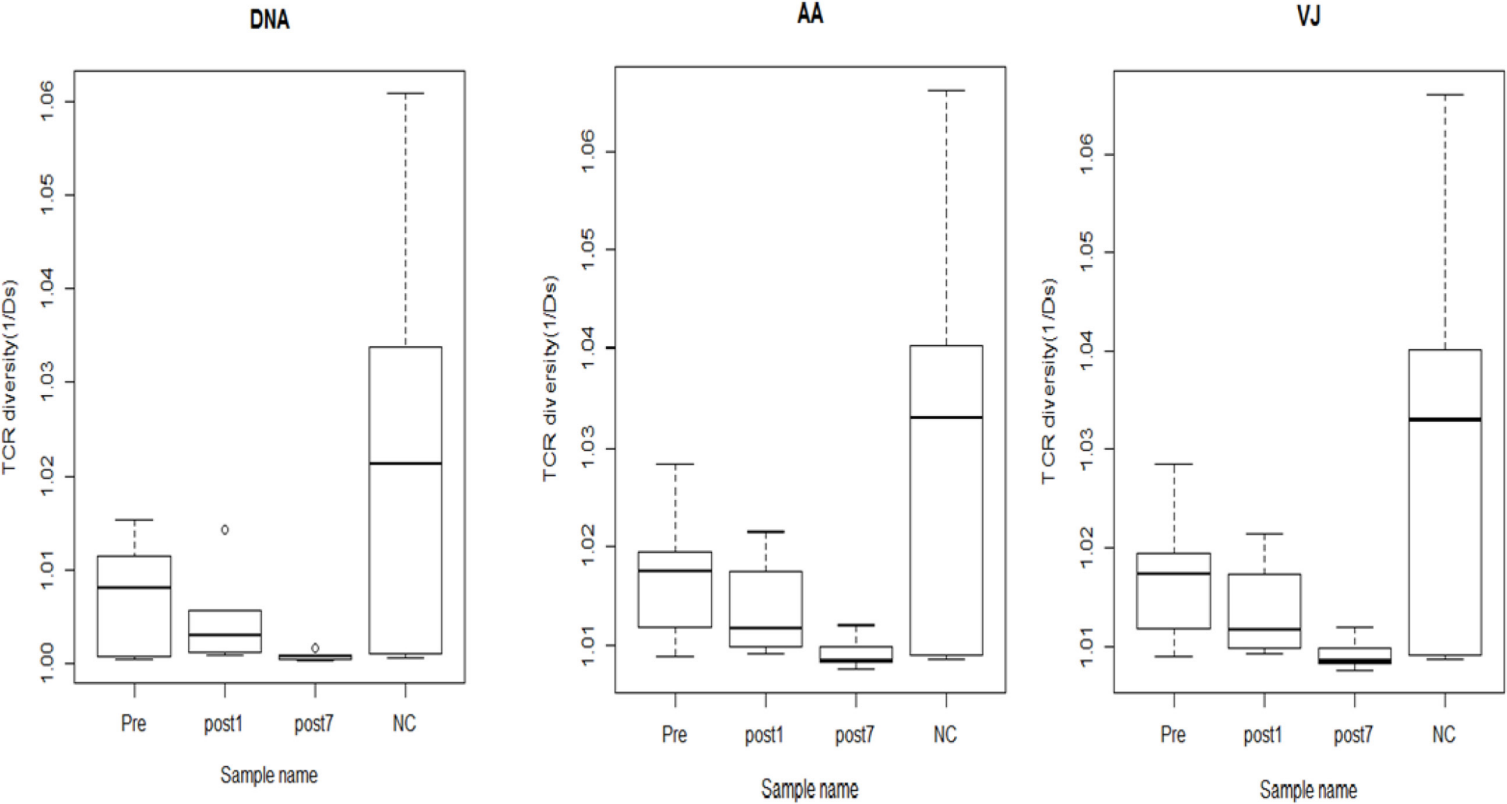
The TCR diversity of the Pre group (*n* = 6), Post1 group (*n* = 6), Post7 group (*n* = 6) and NC group (*n =* 6) (average of six individuals) Error bars, 95% confidence intervals (CI). The TCR diversity of each group was calculated at different resolutions of distinct DNA sequence, amino acid sequence and V-J combination.

To quantify the overall diversity of TCR repertoires of the NC, Pre, Post1 and Post7 groups, we applied the normalised inverse Simpson’s diversity index (1/Ds) to quantitatively measure the diversity performed on DNA sequences, amino acid sequences and V-J combinations. The inverse Simpson’s index (1/Ds) ranges from 1 to ∞, in which 1 indicates no diversity and ∞ indicates the most diversity and is the highest when all clonotypes are equally distributed. The index of the Post7 samples (1/Ds = 1.001 at the DNA level; 1.009 at the amino acid and V-J pair level) was obviously less than that of the Pre (1/Ds = 1.007 at the DNA level; 1.017 at the amino acid and V-J pair level), Post1 (1/Ds = 1.005 at the DNA level; 1.014 at the amino acid and V-J pair level) and NC samples (1/Ds = 1.023 at the DNA level; 1.031 at the amino acid and V-J pair level), and the NC group exhibited the highest TCR diversity (Figure 4). Therefore, our results showed that the T cell TCR repertoire of peripheral blood has much more skewed composition in the Post7 group than in the Post1, Pre- and NC groups, and the complexity of the TCR repertoire tended to decrease postoperatively.

### Comparison of TRβV and TRβJ repertoires between groups

**Figure 5:**
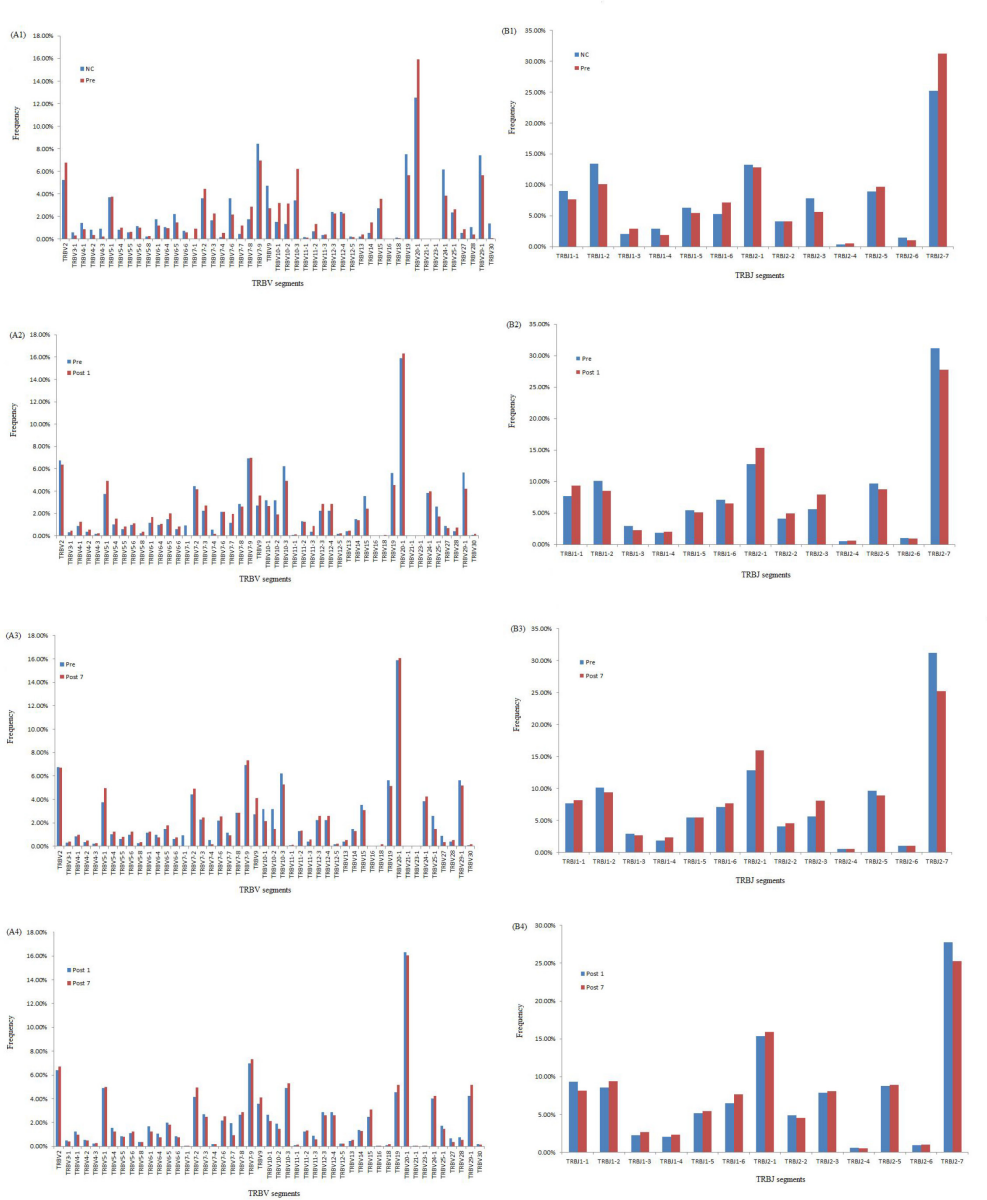
Comparison of TRBV gene and TRBJ gene usage in Pre, Post1, Post7 and NC groups (**A1**) Comparison of TRBV gene usage between NC and Pre groups. (**A2**) Comparison of TRBV gene usage between Pre and Post1 groups. (**A3**) Comparison of TRBV gene usage between Pre and Post7 groups. (**A4**) Comparison of TRBV gene usage between Post1 and Post7 groups. (**B1**) Comparison of TRBJ gene usage between NC and Pre groups. (**B2**) Comparison of TRBJ gene usage Pre and Post1 groups. (**B3**) Comparison of TRBJ gene usage between Pre and Post7 groups. (**B4**) Comparison of TRBJ gene usage between Post1 and Post7 groups.

**Figure 6:**
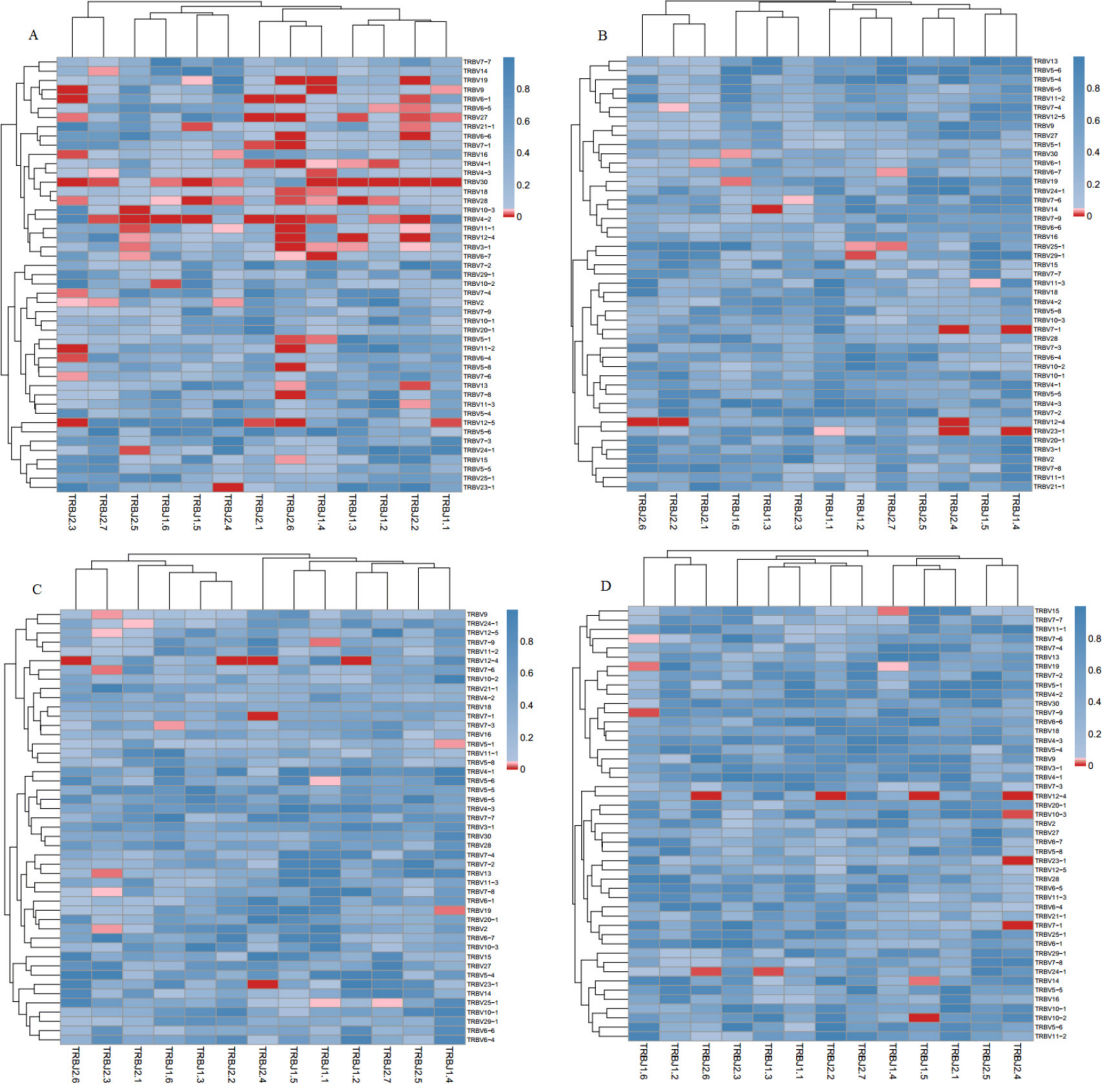
Comparison of the average V–J gene utilization of the sequenced TCRsequences among Pre、Post1、Post7 and NC groups J gene segments are arranged on the x-axis, and V gene segments are arranged on the y-axis. Significant differences were statistically analyzed by the *F*-test. (**A**) Comparison of V-J combinations between Pre and NC groups; (**B**) Comparison of V-J combinations between Pre and Post1 groups; (**C**) Comparison of V-J combinations between Pre and Post7 groups; (**D**) Comparison of V-J combinations between Post1 and Post7 groups. Different colors (blue to red rectangular bands) indicate different levels of significance. (For interpretation of the references to color in this figure legend, the reader is referred to the web version of this article).

We compared the expression levels of the respective TRBV and TRBJ repertoires use frequencies of the Pre group, Post1 group, Post7 group and NC group to analyze whether there is disease specific difference. For the 47 TRβV gene segments, TRBV2, TRBV10-3 and TRBV20-1 were highly expressed in Pre, Post1and Post7 group PBMCs while TRBV30 is significantly poorly expressed in Pre, Post1 and Post7 group PBMCs. For the 13 TRBJ genes segments, TRBJ1-2 showed significantly lower usage in the PBMCs of the Pre, Post1 and Post7 group, whereas TRBJ2-7 is highly expressed in Pre group than in other groups PBMCs (Figure 5). Then, we also compared the relative frequencies of V–J combinations among the four groups. For the TCR repertoires of all subjects (*n* = 24), we were obtained 8159 annotated V–J pairs; among these, we found that the relative frequencies of 104 V–J pairs were significantly different in the Pre group compared to the NC group, 45 V–J pairs were significantly different in the Post1 group compared to the Pre group and there were 32 V–J pairs were significantly different in the Post7 group compared to the Post1 group (Figure 6). Significant differences were considered at *p* < 0.05 by the *t*-test.

## DISCUSSION

Liver transplantation is a well-established modality for treating patients with end-stage hepatitis B cirrhosis for which there are no alternative treatments [15]. The diversity of the immune repertoire is the foundation of the immune response, in which the T cells play an important role. The decreased diversity of the immune repertoire after liver transplantation increases the risks for infection and malignancy [16]. Here, we used next-generation sequencing to investigate the characteristics and polymorphisms of the T-cell receptor complementary-determining region 3 (TCR CDR3) gene using T cells from 6 patients at various time points: preoperative patient group (Pre), 1-day postoperative patient group (Post1) and 7-day postoperative patient group (Post7). We assessed the distribution of CDR3 lengths, VD indel and DJ indel lengths among the 238310735 total good sequences. We observed that the CDR3 length distribution, VD indel and DJ indel lengths were similar among Pre, Post1, Post7 and NC groups. Some researchers have found substantial differences in the distributions of CDR3 lengths between the native and memory T cell pools. It is known that different rearrangements may lead to variable CDR3 lengths, and the characteristics of TCR clonality in different subfamilies can be determined by measuring lengths of CDR3 subfamilies. There are some studies reporting that CDR3 is expressed by CD4 single-positive cells in the thymus [17]. However, the details about the differences and functions of the longer and shorter CDR3 groups need to be intensively studied.

In this study, we used a high-throughput immune sequencing technique to study the diversity of the T-cell repertoire in liver transplantation patients. An interesting observation was that most T cell clones were at very low frequencies (<0.5% of total TCR sequences), which implied that these T cell clones had not undergone clonal expansion. In contrast, a few HECs were present in every individual, which may have resulted from physiological responses to environmental antigens or pathogens. The HECs were greater in the Pre-group than the other groups, while the least was in Post7. A possible explanation for this observation is that newly transplanted livers and the use of the immunosuppressive agents mainly affect the activation and cloning of T lymphocytes of the receptors, which ultimately affects the number of T lymphocytes and the function of the T and B lymphocytes. There are studies indicating that intracellular adenosine triphosphate (iATP) has some value in the immune status, predicting rejection or excessive immune risk [18–19]. These T cells may be gradually cleared by apoptosis 7 days after transplantation. The reduction of donor-reactive clones in tolerant subjects is consistent with our hypothesis that donor-reactive T cells are slowly deleted in response to repeated encounters with donor antigens on quiescent, accepted allografts [20], which results in immune tolerance. The diversity of the immune repertoire is vitally important for health. The greater number of subtypes of immune proteins increases the power of the immune system, and vice versa. We found that the T cell repertoire of the Pre-group and Post1 group was relatively restricted, which may be related to pathology. The Post7 group exhibited the lowest TCR diversity, which is primarily due to the administration of immunosuppressive agents. Therefore, the complexity of this patient’s T cell repertoire did not improve between day 1 and 7 days after transplant. Strategies that improve T cell reconstitution and the recovery of high TCR diversity could greatly reduce transplant-associated morbidity and mortality [21].

There were shared TCR DNA sequences and amino acid sequences between different individuals in each group, and the same TCR sequences existed in more than one individual, also responding to the same antigenic epitope, which was generally considered as an unusual phenomenon [22]. We found 2 amino acid sequences shared by patients in different groups. For example, the amino acid sequence CASSLGGSYEQYF was observed in all Pre, Post1 and Post7 group patients but not in the NC group, and another amino acid sequence, CASSLGETQYF, was observed in Post1, Post7 and NC groups but not in the Pre-group, which may provide biomarkers for transplantation antigens. Thus, a TCR amino acid sequence with suitable antigen-binding characteristics may be encoded in different cells by different nucleotide sequences and selected independently.

Moreover, we further study the characteristics of the repertoire, also systematically analyzed the V, D and J segment usage frequencies in PBMCs at different time points after liver transplantation. For the 47 TRBV genes, TRBV2, TRBV10-3, TRBV20-1 and TRBV30 showed significant different in the comparison of Pre, Post1, Post7 and NC group. TRBJ2-7 gene was highly expressed in Pre group than other groups in the study of 13 TRBJ genes, which suggests that at different times following liver transplantation may result in the expansion or deletion of some T cell populations. We know that under normal circumstances, the immune system is unaffected by any antigen stimulation, the different usage of TRBV and TRBJ genes are rearranged randomly, and T cells show a positive polyclonal state. But in certain disease situation, stimulation by specific antigens can cause the targeted rearrangement and excessive abnormal cloning of one or a few TCR BV subfamilies, then the dominant form of the cloned T cell may suppress the cloning of other T cells, which may result in decreased immune function [23–24].

In conclusion, with the usage of high throughput immune repertoire sequencing technique we succeeded in studying the the entire diversity of the immune repertoire at sequence-level resolution. It could accurately monitor immunosuppressive therapies and responses to infections of transplant patients. But regarding the small sample amount, further investigation should concentrate on the study of immunological sequelae observed in disease states, such as after liver transplantation, potential therapeutic targets and disease biomarker.

## MATERIALS AND METHODS

### Patents and healthy controls

Whole blood samples were collected from 6 patients with end-stage hepatitis B cirrhosis (2 females and 4 males with a mean age of 48.7 ± 6.3 years) and 6 healthy donors (NC,3 females and 3 males with a mean age of 44.2 ± 5.9 years) between 2013 and 2014, and peripheral blood mononuclear cells (PBMCs) were separated. Patients were from the Nephrology Department (organ transplantation and dialysis centre of PLA) at the 181st Hospital, Guangxi, China. All patients underwent liver transplantation surgery, and the patients with end-stage hepatitis B cirrhosis were confirmed by pathologic diagnosis and clinical evidence. The 6 NC were from the Physical Center of the 181st Hospital of Guilin. Whole blood samples were collected from individual healthy donors (*n* = 6) and patients (*n =* 6) at different time points, including before liver transplantation and post-transplant at two time points (post1 and post7), into an ethylenediaminetetraacetic acid (EDTA) VACUTAINERTM blood collection tube (BD, Franklin Lakes, NJ, USA). The clinical diagnosis and pathological features of each patient and the clinical data were reviewed. All the peripheral whole blood samples were collected after the written informed consent was obtained from all subjects or their guardians. The protocol for this research project was approved by the local Ethics Committee.

### T cell isolation, DNA extraction and storage

Peripheral whole blood samples were drawn from elbow vein blood on an empty stomach in the morning from patients and normal controls. PBMCs were prepared from whole blood using lymphocyte separation medium by density gradient centrifugation, and then T cells were isolated from PBMCs by magnetic beads [25]. DNA was extracted from 0.5∼2 × 106 T cells from each sample, using GenFIND DNA (Agencourt, Beckman Coulter, Brea, CA, USA) extraction kits, according to the manufacturer’s instruction. The DNA was transferred to frozen storage tubes and stored at −80° C until used.

T-cell receptor β-chain complementarity determining region 3 (TCRβ CDR3) was defined according to International Immunogenetics collaboration [26], which began with the second conserved cysteine encoded by the 39th AA of the V gene segment and concluded with the conserved phenylalanine encoded by the 59th aa of the J gene segment. We applied Multiplex-PCR to amplify rearranged TCR CDR3 regions from genomic DNA using a set of primers that we specially developed, which contained the forward and reverse primers, respectively, specific to CDR3 Vβ region and Jβ region. The forward and reverse primers and their sequences, at their 5′ ends, were compatible with a GA2 cluster station solid-phase PCR [27]. After the samples were amplified and separated by agarose gel electrophoresis, the products were purified using the QIAquick PCR Purification Kit. The final library was quantitated by two ways: applying the Agilent 2100 bioanalyser instrument (Agilent DNA 1000 Reagents) and real-time quantitative PCR (qPCR) (TaqMan Probe) to determine the average molecule length. The libraries were amplified by the cBot to generate clusters on the flow cell, and an amplified flow cell was pair-end (PE) sequenced using a HiSeq2000 instrument (Illumina, California), using a typical read length of 100 bp.

### High-throughput sequencing and data analysis

The PCR products were sequenced by an Illumina Genome Analyzer, and the quality of raw data that we gained from HiSeq sequencing ranged from 0 to 40. The quality was used to filter out low-quality reads. When we received the data from the sequencer, we first filtered the raw data, including removing adapter contamination. Reads with average quality scores less than 15 (Illumina 0–41 quality system) were removed, the proportion of N bases were lower than 5%, while some bases of the quality lower than 10 were trimmed to ensure the quality score over 15 and that the remaining sequence length was not less than 60 nt. Second, after the filtering, PE read pairs were merged into one contig sequence by two steps: one, aligning the tail parts of two sequences and assessing the identity (software COPE v1.1.3 developed by BGI); the merging required at least 10 bases overlap and had 90% bases matching in the overlapped section; two, as different primers might isolate different length sequences, some of them might be very short, such as less than 100 bp, and will go through all bases on the sequence, which were merged by aligning the head part of the sequences (the software FqMerger developed by BGI). At last, the merged contig sequences and the length distribution plot of the contigs were obtained.

Second, the alignment of TCR data used miTCR software, which was developed by MiLaboratory: http://mitcr.milaboratory.com/downloads/ and the program is automated to adjust the mechanism for errors introduced by sequencing and PC, and can provide alignment statistic information such as CDR3 expression and indels. After alignment, we used the following method for the sequence structural analysis: 1, we counted the numbers of each nucleotide and analysed the proportion at each position; 2, we obtained the indels introduced by V-D-J recombination according to the last position of the V gene on the sequence, the start position of the D gene, the last site of the D gene and the start site of the J gene; 3, the nucleotides were translated into amino acids. In addition, the CDR3 region of each sequence was determined after alignment.

Finally, the expression level of each clone was clear and calculated according to the identity of each sequence after alignment and gained the expression of each distinct DNA sequence, amino acid sequence and V-J combination. Meanwhile, to measure the diversity of each sample, we calculated the distinct clone number, Simpson coefficient and Shannon-Waver coefficient based on different resolution of distinct DNA sequences, amino acids and V-J combinations. The expression level of each sample was also calculated on different resolutions of distinct DNA sequences, amino acids and V-J combinations. In addition, according to the V-J combination profile, the heat-map was plotted. We calculated the TCR repertoire diversity based on the Shannon–Wiener index (H’) [28, 29] and the Simpson index of diversity (Ds) [30].

### Statistical analysis

The biochemical data were expressed as the mean ± standard deviation. The analysis of differences in TCR repertoire among the different groups (including Pre, Post1, Post7 and NC) was performed with a *t*-test. All the statistical analyses were conducted with SPSS 19.0 statistical software and R software and a *p* value < 0.05 was considered significant.

## SUPPLEMENTARY MATERIALS TABLES







## Abbreviations

CDR: complementarity-determining region
D: Diversity
Ds: Simpson index of diversity
HBV: Hepatitis B virus
H’: Shannon–Wiener index
HECs: highly expanded clone
IR: Immune repertoire
J: Joining
MHC: major histocompatibility complex
NC: healthy controls
PBMCs: peripheral blood mononuclear cells
Pre: 1 days before transplantation
Post1: first after transplantation
Post7: on the seventh days after transplantation
TCR: T cell receptor
V: variable
