# Correction: Characteristic analysis of TCR β-chain CDR3 repertoire for preand post-liver transplantation

**DOI:** 10.18632/oncotarget.27560

**Published:** 2020-05-05

**Authors:** Guiqi Yang, Minglin Ou, Huaizhou Chen, Changchun Guo, Jiejing Chen, Hua Lin, Donge Tang, Wen Xue, Wenlong Li, Weiguo Sui, Yong Dai

**Affiliations:** ^1^ Guangxi Key laboratory of Metabolic Diseases Research, Central Laboratory of Guilin No. 181 Hospital, Guilin 541002, Guangxi, P.R. China; ^2^ Clinical Medical Research Center, The Second Clinical Medical College of Jinan University, Shenzhen, Guangdong 518020, P.R. China; ^3^ The Pingshan People’s Hospital of Shenzhen, Shenzhen, Guangdong 518118, P.R. China; ^4^ The Technology Company of iCarbonX, Shenzhen, Guangdong 518000, P.R. China

**This article has been corrected:** The 1st affiliation information was presented incorrectly. The proper affiliation 1 is as follows:

^1^Guangxi Key laboratory of Metabolic Diseases Research, Central Laboratory of Guilin No. 181 Hospital, Guilin 541002, Guangxi, P.R. China

Original article: Oncotarget. 2018; 9:34506–34519. 34506-34519. https://doi.org/10.18632/oncotarget.26138

