# Supplementary material for: Characteristic analysis of TCR β-chain CDR3 repertoire for pre- and post-liver transplantation
Source: Oncotarget. 2018 Oct 2;9(77):34506–19. doi: 10.18632/oncotarget.26138 (PMC6195376; doi:10.18632/oncotarget.26138)
Supplement: Supplementary file 1 [file oncotarget-09-34506-s001.pdf]

Characteristic analysis of TCR β-chain CDR3 repertoire for pre- and post-liver transplantation

SUPPLEMENTARY MATERIALS

Supplementary Table 1: DNA sequence of highly expanded clones. See Supplementary_Table_1

Supplementary Table 2: A summary of the public CDR3 sequences in NC, Pre, Post1, and Post7 groups. See Supplementary_Table_2
