# Supplementary material for: Characteristic analysis of TCR β-chain CDR3 repertoire for pre- and post-liver transplantation
Source: Oncotarget. 2018 Oct 2;9(77):34506–19. doi: 10.18632/oncotarget.26138 (PMC6195376; doi:10.18632/oncotarget.26138)
Supplement: Supplementary file 2 [file oncotarget-09-34506-s002.doc]

Supplementary Table 1: DNA sequence of highly expanded clones

| Sanple | clonotype | Frequency (%) |
| --- | --- | --- |
| Pre-1 | TGCGCCAGCAGTAAAGGGGGGGTTATGGAGACCCAGTACTTC | 9.28 |
|  | TGTGCCAGCGTACGTCTTGGCAAGCAGTACTTC | 2.89 |
|  | TGTGCCAGCAGCTTAGTGGGGGGGCGGAACACTGAAGCTTTCTTT | 2.32 |
|  | TGTGCCAGCAGCTTCACTAATACTATGGCTACACCTTC | 1.86 |
|  | TGCAGTGCTAGTCGATAGACTGAACACTGAAGCTTTCTTT | 1.72 |
|  | TGTGCCAGCAGCCAAGATCGGACCAGGCAAGCTTTCTTT | 1.27 |
|  | TGTGCCAGCTATGGGTCTTCAGGATCGGGACATTATAATTCACCCCTCCACTTT | 0.95 |
|  | TGTGCCAGCACGGCCGGCGACACCATATATTTT | 0.93 |
|  | TGTGCCAGCAGCAATCAGCGCCAGGGCATAGGGTACGAGCAGTACTTC | 0.65 |
|  | TGCAGCGTTGAAACTTACTTCGAAGAGACCCAGTACTTC | 0.55 |
| Pre-2 | TGCAGTGCCCCAGATGACACAGCCAACGTCCTGACTTTC | 1.32 |
|  | TGTGCCAGTAGAAGCAGGGGGGCGGATACTGAAGCTTTCTTT | 0.90 |
|  | TGCAGTGCTAGAGGACAGGGTGGCAATCAGCCCCAGCATTTT | 0.79 |
|  | TGTGCCAGCAGCTGGGGTAACGAGCAGTACTTC | 0.75 |
|  | TGTGCCAGCAGCCAACAGGGTGCGACAGGATCACCCCTCCACTTT | 0.65 |
|  | TGTGCCAGCAGGTCCGGGACAGGGGGCACAGCACCCCTCCACTTT | 0.58 |
| Pre-3 | TGTGCCATCAGGCTTCCTCACTGCGGGACGACAATGAGCAGTTCTTC | 1.48 |
| Pre-4 | TGCGCCAGCAGTGAGAGGGGTCGTCCTACGAGCAGTACTTC | 8.07 |
|  | TGTGCCACCAGCAGAGCTGGGGAGGGCCGGGATGAGCAGTTCTTC | 3.79 |
|  | TGTGCCAGCAGTTCTGGGAATGAGCAGTTCTTC | 2.68 |
|  | TGTGCCAGCAGCTTAGATTCATGTCACAGTCTACGGAAACACCATATATTTT | 2.27 |
|  | TGTGCCAGCAGCTTAGGCGGATTATGGGAGCAGTACTTC | 1.77 |
|  | TGTGCCAGCAGTGACGGTATGAGCAGTTCTTC | 1.58 |
|  | TGTGCCAGCAGTAAGATTAATTCACCCCTCCACTTT | 1.49 |
|  | TGTGCCAGTAGTCGGACAGGGGAATCCTACGAGCAGTACTTC | 1.16 |
|  | TGTGCCAGCAGTGAAACGGGATCGGGCTACGAGCAGTACTTC | 1.08 |
|  | TGTGCCAGCAGCTTAGGGACAGGAGCTCCTACGAGCAGTACTTC | 0.91 |
|  | TGCAGTGCCGCCCTAGCGGGGGAGCAAGAGACCCAGTACTTC | 0.90 |
|  | TGTGCCAGCAGTGAGACGGAGACCCAGTACTTC | 0.78 |
|  | TGTGCCAGCAGCTTCTTAACAGGGTAAGAGACCCAGTACTTC | 0.75 |
|  | TGTGCCACCAGCAGAGGCGAGGGGCGGGATACGCAGTATTTT | 0.59 |
|  | TGTGCCACCGCCCCGGGCCTCTCCTACGAGCAGTACTTC | 0.56 |
|  | TGTGCCAGCAGCTTAGACTATGGAGCGGGAGGGCCGGGGAATGAGCAGTTCTTC | 0.55 |
| Pre-5 | TGTGCCACCGCCCCGGGCCTCTCCTACGAGCAGTACTTC | 5.43 |
|  | TGTGCCATCTTGGGAGGGATAAATTACGAGCAGTACTTC | 1.08 |
|  | TGTGCCAGCAGCCCTTCCTATGGCTACACCTTC | 0.95 |
|  | TGTGCCAGCAGCCCACCGACAGGTGACTACGAGCAGTACTTC | 0.91 |
|  | TGTGCCAGCAGCTTAGGACAGGGGATCTACAACGTCCTGACTTTC | 0.81 |
|  | TGTGCCAGCAGCCAAGATCGGCAGACCCAGTACTTC | 0.58 |
|  | TGTGCCAGCAGCAAAACCGGGACAGGGGTCTACGAGCAGTACTTC | 0.53 |
|  | TGTGCCAGCAGCCCCCCAACTGGCTCCTACGAGCAGTACTTC | 0.52 |
| Pre-6 | TGCAGTGCAAGAACTAGCGGGAGGGCGTCCGGGCGAGCAGTACTTC | 12.02 |
|  | TGTGCCAGCAGTTCCTGGGACAGGGACAATTCACCCCTCCACTTT | 2.09 |
| Post1-1 | TGTGCCAGCAGCTTAGTGGGGGGGCGGAACACTGAAGCTTTCTTT | 7.51 |
|  | TGCAGTGCTAGTCGATAGACTGAACACTGAAGCTTTCTTT | 5.52 |
|  | TGTGCCAGCAGCGAAGCCGCGGTGACGACAGATACGCAGTATTTT | 3.03 |
|  | TGTGCCAGCTATGGGTCTTCAGGATCGGGACATTATAATTCACCCCTCCACTTT | 2.87 |
|  | TGTGCCAGCGTACGTCTTGGCAAGCAGTACTTC | 2.59 |
|  | TGCGCCAGCAGTAAAGGGGGGGTTATGGAGACCCAGTACTTC | 2.35 |
|  | TGTGCCAGCAGCTTCACTAATACTATGGCTACACCTTC | 2.28 |
|  | TGCAGTGCTCCGCGGGGACCTAATGAGCAGTTCTTC | 2.26 |
|  | TGTGCCAGCAGCTTAGATCTCCGCGGGGAGCAGTTCTTC | 2.11 |
|  | TGTGCCAGCACCTATGGGGTTGGGGCCTACGAGCAGTACTTC | 1.71 |
|  | TGTGCCAGCAGCAATCAGCGCCAGGGCATAGGGTACGAGCAGTACTTC | 0.82 |
|  | TGTGCCAGCAGTTTAGAGCAAGGAGCGGGAGAGACCCAGTACTTC | 0.70 |
|  | TGTGCCACCGCCCCGGGCCTCTCCTACGAGCAGTACTTC | 0.57 |
| Post1-2 | TGTGCCAGCAGCTGGGGTAACGAGCAGTACTTC | 1.12 |
|  | TGTGCCAGCAGTGACATGGAGGGGGGTTCACCCCTCCACTTT | 0.60 |
|  | TGCAGTGCCCCAGATGACACAGCCAACGTCCTGACTTTC | 0.57 |
|  | TGTGCCAGTAGAAGCAGGGGGGCGGATACTGAAGCTTTCTTT | 0.56 |
|  | TGCAGTGCTAGAGGACAGGGTGGCAATCAGCCCCAGCATTTT | 0.56 |
|  | TGTGCCAGCAGCCAAGGAGAGGGCCTCGCAGATACGCAGTATTTT | 0.55 |
|  | TGTGCCAGCAGCCTCGGACAGGGGGTCTACGAGCAGTACTTC | 0.51 |
| Post1-3 | TGTGCCATCAGGCTTCCTCACTGCGGGACGACAATGAGCAGTTCTTC | 2.89 |
|  | TGTGCCAGCACCGGGACTGTCGTACTACGAGCAGTACTTC | 0.55 |
| Post1-4 | TGCGCCAGCAGTGAGAGGGGTCGTCCTACGAGCAGTACTTC | 5.45 |
|  | TGTGCCACCAGCAGAGCTGGGGAGGGCCGGGATGAGCAGTTCTTC | 1.17 |
|  | TGTGCCAGCAGCTTAGGCGGATTATGGGAGCAGTACTTC | 0.97 |
|  | TGTGCCAGCAGCTTAGATTCATGTCACAGTCTACGGAAACACCATATATTTT | 0.69 |
|  | TGTGCCAGCATTCTACTGAAGCTTTCTTT | 0.67 |
|  | TGTGCCAGTAGTCGGACAGGGGAATCCTACGAGCAGTACTTC | 0.65 |
|  | TGTGCCAGCAGTCAAGGTTCGGACACCGGGGAGCTGTTTTTT | 0.60 |
| Post1-5 | TGTGCCATCAGTGCGATGGGGGCCGATTCACCCCTCCACTTT | 0.68 |
|  | TGTGCCTCCCGAGGAGCTGCGAACACCGGGGAGCTGTTTTTT | 0.68 |
|  | TGTGCCAGCAGTGACTGGGGTACAAATTCACCCCTCCACTTT | 0.61 |
|  | TGCGCCAGCAGTGAGTCGGGTGGATACAATGAGCAGTTCTTC | 0.55 |
|  | TGTGCCATCAACGGGGAACACTGAAGCTTTCTTT | 0.55 |
|  | TGTGCCACCAGTGATTTGCCCCCGGGCCAAGGTGATACGCAGTATTTT | 0.54 |
|  | TGCGCCAGCAGCTTATTGGGACTCGATGGCTACACCTTC | 0.52 |
|  | TGCAGTGCCACCCCGGACAAGAACACTGAAGCTTTCTTT | 0.51 |
|  | TGTGCCAGCAGTGAACCTGACTAGCGGGTAACCGGGGAGCTGTTTTTT | 0.51 |
|  | TGTGCCACCAGTGAGGCGCTGACAGGGTTAGGAGAGACCCAGTACTTC | 0.51 |
| Post1-6 | TGCAGTGCAAGAACTAGCGGGAGGGCGTCCGGGCGAGCAGTACTTC | 7.16 |
|  | TGTGCCAGCAGTTCCTGGGACAGGGACAATTCACCCCTCCACTTT | 1.26 |
| Post7-1 | TGCGCCAGCAGTAAAGGGGGGGTTATGGAGACCCAGTACTTC | 1.62 |
|  | TGTGCCAGCAGCTTAGTGGGGGGGCGGAACACTGAAGCTTTCTTT | 0.70 |
| Post7-2 | TGTGCCAGCAGCTGGGGTAACGAGCAGTACTTC | 1.59 |
|  | TGTGCCAGCAGGTCCGGGACAGGGGGCACAGCACCCCTCCACTTT | 1.52 |
|  | TGTGCCAGTAGAAGCAGGGGGGCGGATACTGAAGCTTTCTTT | 0.69 |
|  | TGTGCCAGCTACCACCATGCGGGGGTGGGCTATGGCTACACCTTC | 0.68 |
|  | TGCAGTGCCCCAGATGACACAGCCAACGTCCTGACTTTC | 0.57 |
| Post7-3 | TGTGCCATCAGGCTTCCTCACTGCGGGACGACAATGAGCAGTTCTTC | 2.80 |
|  | TGTGCCACCGCCCCGGGCCTCTCCTACGAGCAGTACTTC | 1.04 |
| Post7-4 | TGCGCCAGCAGTGAGAGGGGTCGTCCTACGAGCAGTACTTC | 2.16 |
|  | TGTGCCAGCAGCTTAGGCGGATTATGGGAGCAGTACTTC | 0.77 |
|  | TGTGCCACCAGCAGAGCTGGGGAGGGCCGGGATGAGCAGTTCTTC | 0.61 |
| Post7-5 | TGCAGTACGTAAGGACAGGGCCTTCCGCTACGAGCAGTACTTC | 0.51 |
| Post7-6 | TGCAGTGCAAGAACTAGCGGGAGGGCGTCCGGGCGAGCAGTACTTC | 0.78 |
| NC-1 | TGTGCCAGTACTAAGGGGGTTGGATTAGAGACCCAGTACTTC | 13.01 |
|  | TGTGCCAGTAGTATCTCCCAAGACACTGAAGCTTTCTTT | 3.79 |
|  | TGTGCCAGTAGTATATCTCAGGACACTGAAGCTTTCTTT | 3.45 |
|  | TGTGCCAGTAGTATAAGTCAAGACACTGAAGCTTTCTTT | 2.99 |
|  | TGCAGTGCCATAAAACCGGGACTAGGGCTACGAGCAGTACTTC | 2.41 |
|  | TGTGCCAGCAGCCACAACAGGGGGATACAAACTGAAGCTTTCTTT | 1.71 |
|  | TGTGCCAGCAGTCCAGGGACAGGGCCCTACGAGCAGTACTTC | 1.53 |
|  | TGTGCCAGCAGCCCAACAGGGGGCACTGAAGCTTTCTTT | 1.37 |
|  | TGTGCCAGCAGCTCGACAGGGGTGGATTCGGATACGCAGTATTTT | 1.34 |
|  | TGCAGCGTGAGGGCTGGCCAAGAGACCCAGTACTTC | 1.30 |
|  | TGTGCCAGCAGCTCCTCAGAGGGGGACAGAGCTAACGTTTATGGCTACACCTTC | 1.26 |
|  | TGCAGTGCTTATTACCGCCCCAGCTTCAACTACGAGCAGTACTTC | 1.24 |
|  | TGTGCCAGCAGCTTGGTGTTGTCGGGGGAGCAGTTCTTC | 0.88 |
|  | TGTGCCAGCAGCCCCACGAGCAGTTCGAACTATGGCTACACCTTC | 0.63 |
| NC-2 | TGCAGCGTTGGACTCAATGGGCGCGAGCAGTACTTC | 17.23 |
|  | TGTGCCACCAGTGATGGCGACTGACAGGAGGGCTACGAGCAGTACTTC | 4.58 |
|  | TGCGCCAGCAGTGAGTCGGACTCTGGGGCCAACGTCCTGACTTTC | 1.78 |
|  | TGTGCCAGCAGTTACTCGGGGGCGGACGAGCAGTACTTC | 0.94 |
|  | TGTGCCACCAGTGATTTGCCGGATCCACTTT | 0.85 |
|  | TGTGCCAGCAGCTTAGAGGCCGTTTCCTACAATGAGCAGTTCTTC | 0.56 |
|  | TGTGCCAGCAGTGAAGACCCCCGGCCCTCGACTGCGGATGGCTACACCTTC | 0.56 |
|  | TGTGCCAGCAGCGCCCACGGGGCGGGGGCCAACGTCCTGACTTTC | 0.56 |
|  | TGTGCCAGCAGTTACTCGGGCTCCGACGAGCAGTACTTC | 0.54 |
|  | TGTGCCAGCAGTCAATTTTAGGACCCTGGACTAGCGGTTGAGCAGTTCTTC | 0.54 |
| NC-3 | TGTGCCACCAGCAGAGATACGGCAGGCACTGAAGCTTTCTTT | 2.63 |
|  | TGCAGTGCTAGAGGGGACTCGGGGATCTACGAGCAGTACTTC | 0.90 |
|  | TGCAGTGTCCGGGGACAGGCCTACGAGCAGTACTTC | 0.71 |
|  | TGCGCCAGCAGCTTGGCTAGGGCAGGGGGCGCGTCTGGCTACACCTTC | 0.52 |
| NC-4 | TGTGCCAGTAGCCCTGCCAGCTCCTACAATGAGCAGTTCTTC | 0.90 |
|  | TGTGCCATCAGTGATCTGGGGGGTACCTACGAGCAGTACTTC | 0.80 |
|  | TGTGCCAGCAGCTTGGTAGGACAGGAGAACGAGCAGTACTTC | 0.59 |
|  | TGCGCCAGCAGTGAAGAGGGCTATAGCAATCAGCCCCAGCATTTT | 0.56 |
|  | TGTGCCAGCACCCGGGCGGACGTTGTCATCAAGGAGCACAGATACGCAGTATTTT | 0.53 |
| NC-5 | TGTGCCAGCAGCTCGCAGAACTATGGCTACACCTTC | 23.27 |
|  | TGTGCCAGCACAGACAGGGACTCTCCTACGAGCAGTACTTC | 4.36 |
|  | TGCGCCAGCAGCTTGGCGGGGACAGGGGTGATTCACCCCTCCACTTT | 2.93 |
|  | TGTGCCAGCAGCCCCGGGGGGGGGACCTACGAGCAGTACTTC | 1.00 |
|  | TGTGCCAGCAGCGCCATCCTCCTACGAGCAGTACTTC | 0.84 |
|  | TGTGCCAGTAGTATGGCGGGCAATGAGCAGTTCTTC | 0.63 |
|  | TGTGCCAGCAGCCAAGATGACGGAACTGGAAACACCATATATTTT | 0.62 |
| NC-6 | TGTGCCAGCAGTGAGGGGACAGAGACCCAGTACTTC | 9.81 |
|  | TGTGCCACCAGTGATAGCGGCCATCGTGGGACCTACAATGAGCAGTTCTTC | 7.04 |
|  | TGTGCCTGGAGAGGCGATGAGCAGTTCTTC | 5.97 |
|  | TGTGCCAGTAGTCCCAGGAACTACAATGAGCAGTTCTTC | 1.78 |
|  | TGTGCCAGCAGCGGATCTAGAGAGGGCTCCTACGAGCAGTACTTC | 1.12 |
|  | TGCAGCGTTGACCCCACTTACACTGAAGCTTTCTTT | 1.09 |
|  | TGCAGCGTGGGCGTGGACAGCAACACTGAAGCTTTCTTT | 0.70 |
|  | TGTGCCAGCACCTCCTTTGGGGGAACGAGGGATACGCAGTATTTT | 0.65 |
|  | TGCAGCGTTGGTTCGGACAGCAACACTGAAGCTTTCTTT | 0.55 |
