# Supplementary material for: Characteristic analysis of TCR β-chain CDR3 repertoire for pre- and post-liver transplantation
Source: Oncotarget. 2018 Oct 2;9(77):34506–19. doi: 10.18632/oncotarget.26138 (PMC6195376; doi:10.18632/oncotarget.26138)
Supplement: Supplementary file 3 [file oncotarget-09-34506-s003.docx]

**Supplementary Table 2: A summary of the public CDR3 sequences in NC, Pre, Post1, and Post7 groups**

| Groups | Clonotype | | Public sequences |
| --- | --- | --- | --- |
| NC | | AA | CALE~TIYF |
|  | |  | CASSL~PQHF |
|  | |  | CASSLGGTEAFF |
|  | |  | CASSLG~ETQYF |
|  | |  | CAGLGGRDQETQYF |
|  | |  | CASSLGETQYF |
|  | |  | CANTGELFF |
|  | |  | CAS~YF |
|  | |  | CANYGYTF |
|  | |  | CASSLTDTQYF |
|  | |  | CAPII~PLHF |
|  | |  | CASSLG~TDTQYF |
|  | |  | CALGP~VLTF |
|  | |  | CASSL~ETQYF |
|  | |  | CAASRGC~AKNIQYF |
|  | |  | CASSCAP~NSPLHF |
|  | |  | CAQQRLGGFEVA~GRLCSGNTIYF |
|  | |  | CSCAPI~SPLHF |
|  | |  | CSANYGYTF |
|  | |  | CASSLGTDTQYF |
|  | |  | CASSQETQYF |
|  | |  | CASS~QHF |
|  | |  | CARVPRAV~NTGELFF |
|  | |  | CASSL~GELFF |
|  | |  | CASCAP~NSPLHF |
|  | |  | CASSP~ELFF |
|  | |  | CASSPSTDTQYF |
|  | |  | CASSLGYEQYF |
|  | |  | CASRL*~TDTQYF |
|  | |  | CASSLGTEAFF |
|  | | DNA | TGTGCTCTGGAAACACCATATATTTT |
|  | |  | TGTGCCAGCTGTGCTCCTATAATTCACCCCTCCACTTT |
|  | |  | TGCGCCAGGGTCCCCAGGGCTGTGCGAACACCGGGGAGCTGTTTTTT |
|  | |  | TGTGCCAGCAGCTGTGCTCCTATAATTCACCCCTCCACTTT |
|  | |  | TGTGCGGGGCTCGGGGGCCGTGACCAAGAGACCCAGTACTTC |
|  | |  | TGTGCGAACACCGGGGAGCTGTTTTTT |
|  | |  | TGTGCCTCCAGGCTGTGAGCACAGATACGCAGTATTTT |
|  | |  | TGTGCTCTGGGGCCAACGTCCTGACTTTC |
|  | |  | TGTGCCGCGTCTCGGGGCTGTGAGCCAAAAACATTCAGTACTTC |
|  | |  | TGTGCTCCTATAATTCACCCCTCCACTTT |
|  | |  | TGCAGTGCTAACTATGGCTACACCTTC |
|  | |  | TGCAGCTGTGCTCCTATAATTCACCCCTCCACTTT |
|  | |  | TGTGCTCAGCAGAGACTGGGGGGTTTTGAAGTGGCCCTGGGAGGCTGTGCTCTGGAA |
|  | |  | TGTGCCAGCAGCCCCAGCATTTT |
|  | |  | TGTGCTAACTATGGCTACACCTTC |
| Pre | | AA | CATSDSNQPQHF |
|  | |  | CASSQGQGGYEQYF |
|  | |  | CASSLGNEQYF |
|  | |  | CASSLTGKLFF |
|  | |  | CASSLGYEQYF |
|  | |  | CASSGQQGSNEQFF |
|  | |  | CASRGL^~^NEQFF |
|  | |  | CASSFGDRAYNEQFF |
|  | |  | CASSFGTVNSPLHF |
|  | |  | CASSYSYEQYF |
|  | |  | CATSRVAGETQYF |
|  | |  | CASSLGSSYEQYF |
|  | |  | CASSLGGSYEQYF |
|  | |  | CASSGGYNSPLHF |
| Post1 | | AA | CASSLGETQYF |
|  | |  | CASSLGG^~^SYEQYF |
|  | |  | CASSLDSYEQYF |
|  | |  | CASSLGGNEQFF |
|  | |  | CASSLYNEQFF |
|  | |  | CASSSSYEQYF |
|  | |  | CSVVQHTQYF |
|  | |  | CASSLGYEQYF |
|  | |  | CASSLGQGVYEQYF |
|  | |  | CASSSYNEQFF |
|  | |  | CATSRVAGETQYF |
|  | |  | CASSLGGSYEQYF |
| Post7 | | AA | CASSLGQGPYEQYF |
|  | |  | CASSSSYEQYF |
|  | |  | CASSLVGYEQYF |
|  | |  | CASSLSYEQYF |
|  | |  | CASSLGNQPQHF |
|  | |  | CASSFSYEQYF |
|  | |  | CASSL^~^RGAVF |
|  | |  | CASSQ^~^YEQYF |
|  | |  | CASSQGQGYEQYF |
|  | |  | CASSLG~YEQYF |
|  | |  | CASSLGQ~TYEQYF |
|  | |  | CASSPRGSYEQYF |
|  | |  | CASSPGTAYEQYF |
|  | |  | CASSLGGSYEQYF |
|  | |  | CASSLGETQYF |
|  | |  | CASSLNSNQPQHF |
|  | |  | CAS~YF |
|  | |  | CASSLDSYEQYF |
|  | |  | CASSVGDTQYF |
|  | |  | CASSLGQGYEQYF |
|  | |  | CASSLGVYEQYF |
|  | |  | CSARGGTEAFF |
|  | |  | CASSLG~SYEQYF |
|  | |  | CASSLEGNEQFF |
|  | |  | CAS~FF |
|  | |  | CASSL~ETQYF |
|  | |  | CASSLGSSYEQYF |
|  | |  | CASSLGGEQYF |
